# Evaluation of the Biological Activity of Manna Exudate, from *Fraxinus ornus* L., and Its Potential Use as Hydrogel Formulation in Dermatology and Cosmetology

**DOI:** 10.3390/gels10060351

**Published:** 2024-05-21

**Authors:** Carla Villa, Francesco Saverio Robustelli della Cuna, Elena Grignani, Sara Perteghella, Davide Panzeri, Debora Caviglia, Eleonora Russo

**Affiliations:** 1 Department of Pharmacy, Section of Drug and Cosmetic Chemistry, Viale Benedetto XV 3, 16132 Genoa, Italy; carla.villa@unige.it (C.V.); debora.caviglia@edu.unige.it (D.C.); 2 Environmental Research Center, ICS Maugeri SPA SB, Institute of Pavia, IRCCS, Via Maugeri 2, 27100 Pavia, Italy; saverio.robustelli@icsmaugeri.it (F.S.R.d.C.); elena.grignani@icsmaugeri.it (E.G.); 3 Department of Drug Sciences (DDS), University of Pavia, Via Taramelli 12, 27100 Pavia, Italy; sara.perteghella@unipv.it; 4 Department of Biotechnology and Bioscience, University of Milan-Bicocca, Piazza della Scienza 2, 20126 Milan, Italy; davide.panzeri@unimib.it

**Keywords:** manna, hydrogel formulation, cosmetology, dermatology

## Abstract

Manna, a well-known herbal drug has multiple traditional and pharmaceutical uses and the entire composition, sugar derivatives and polyphenols, gives rise to a very interesting bioactive complex with versatile therapeutic and benefic properties such as antioxidant and anti-inflammatory activities. The aim of this research was to investigate a *F. ornus* manna extract loaded in a pectin hydrogel as a synergic vehicle to evaluate the potential use of the complex for cosmetic and dermatological applications. In particular, the study set out to disclose manna properties as a wound healing agent with antimicrobial and reparative activity on infected tissues. Moreover, considering the correlation between antioxidant activity and antiaging potential, the extract was investigated in regard to the anti-elastase activity and skin whitening potential. The total phenolic content of each extract was also determined and a safe profile by in vitro cytotoxicity studies was verified. The hydrogel complex, containing the manna extract and pectin as the gelling agent, exhibited suitable properties in terms of pH (from 5.50 to 6.80), rheological behavior and ability of preserving the antioxidant activity of the manna exudate (around 10%). All the peculiarities that make the pectin hydrogels ideal systems for skin disease, as wound dressings and for antiaging cosmetic formulations.

## 1. Introduction

In European medicine, and as reported in Italian Pharmacopoeia [1], the herbal drug known as manna is made up of the solidified sugary sap that flows from incisions on the trunk of some ash tree species, namely manna ash, *Fraxinus ornus* L., and *Fraxinus angustifolia Vahl* subsp. *angustiflolia.* Both trees are endemic species of Sicily, which are still cultivated in the Madonie Mountains for the production of manna. In the summertime, each plant can produce from 0.5 to 1.5 Kg of drug depending on the agro-climatic conditions. Depending on the harvesting method, manna can be distinguished into different types, namely “manna in cannoli” (long hollow tubes) almost completely free of impurities, and manna “rottame” or in “sorte” with different degrees of impurities [2]. The history of manna production and harvesting in Sicily is very ancient [3]. The name, manna, would derive from the old Arabic term gibel-el-man (mountain of manna) [4] and its culture or manniculture, developed intensively in the 18th century, mainly in the Madonie Mountains [5], where it is currently practiced in a few restricted areas.

Manna’s most abundant active compound is the hexavalent alcohol D-mannitol, also known as “manna sugar” [6], an osmotically active cell-compatible polyol [5] responsible for the consolidated use of manna as a natural remedy against constipation and as a sweetener. Its activity and safety profile are well known in medicine and pharmacology, but mannitol is also widely used in the food and skincare industries. Its hydrating and antioxidant properties make it an ideal ingredient for use in cosmetic and dermatologic formulations. It has a powerful free radical scavenger activity that protects dermal cell membranes and extracellular matrix components from reactive oxygen species and products of UV radiation [7]. Like many sugars, it binds water molecules and enhances the skin’s moisture-retaining capacity providing volume, firmness, and elasticity. In addition, it can break down bacterial films, a property that makes mannitol useful in acne treatment products.

Other manna constituents are simple sugars (glucose, fructose) (2–3%), complex carbohydrates (mannotriose and mannotetraose) (8–10%), lactic (1%), succinic (0.2%) and malic (0.07%) acids, water (10%) and ash (4%). Small percentages of inorganic compounds such as potassium (0.5%), magnesium (0.06%) and calcium (0.09%) are also present. Fatty acids represent 0.2% of the drug, with a high amount of oleic and palmitic acids [8,9]. Coumarins can be found in both free and glycoside forms, in particular esculetin, fraxetin and lomatin [10]. The peculiar phenolic fraction, consisting of lignans (pinoresinol, siringaresinol) and phenols (tyrosol, dopaol, 4-hydroxy phenylacetic acid), has been proposed as a marker to assess natural product authenticity or adulterations [6].

The entire composition gives rise to a very interesting bioactive complex, whose medicinal properties have been known since ancient times, as evidenced by therapeutic applications in traditional Arabic medicine. Manna drug has multiple traditional and pharmaceutical uses: as a mild laxative, without side effects, for the expulsion of intestinal parasites, and as a regulator of hepatic and intestinal functions [11,12,13]. It is a natural sweetener, low in glucose and fructose, well-tolerated by diabetic patients and largely used by the food industry.

Because of its emollient properties, manna can be used as a valuable ingredient in the preparation of cosmetic creams and soaps [2,14]. Moreover, preliminary data obtained by in vitro tests showed that a manna extract of ash enriched in mannitol could improve epidermal barrier function, reduce inflammation and stimulate the 5α-reductase enzyme [15].

Recently, the application of antioxidant ingredients in skin-lightening cosmetic products has been increasing [16] to counteract the oxidative effect of UV-irradiation in melanogenesis by tyrosinase activation [17]. Considering that the total phenolic and flavonoid contents of natural extracts are strictly correlated with free radical-scavenging and tyrosinase-inhibiting activities, manna complex could be considered as a suitable ROS scavenger candidate to reduce hyperpigmentation [18,19].

Considering all these statements and our interest in natural pharmaceutical and cosmetic ingredients [20,21,22], the purpose of our research was to investigate the beneficial skin properties of a *F. ornus* manna extract and its potential use for cosmetic and dermatological applications. To this end, loading the extract into an appropriate pectin hydrogel as a synergistic vehicle was considered to evaluate the potential use of the complex for cosmetic and dermatological applications.

Hydrogels are three-dimensional networks of hydrophilic polymers that can absorb and retain large water amounts. Their good biocompatibility makes them very attractive and useful in medical applications such as drug delivery systems, in tissue engineering, wound healing, etc. [23].

Pectin is a natural polymer composed of d-galacturonic acid units joined by α-(1→4) glycosidic bond chains [24]. It can be isolated from different natural raw materials (fruit peels and pulp, sugar beet and sunflower heads) affecting composition, molecular weight and dispersibility in water. One of the most important properties of pectin is its gelling ability, forming ionically crosslinked hydrogels in the presence of precursor ions, such as Ca^2+^, Mg^2+^, Zn^2+^.

Physicochemical and biological properties of pectin (e.g., immunoregulatory, antibacterial, antitumor, antioxidant, anti-inflammatory and hypoglycemic activities) combined with the hydrogel characteristics give rise to an attractive strategy for tissue engineering applications. Pectin hydrogels possess several peculiar properties that make them ideal systems for skin diseases and wound dressings, such as good self-healing behavior, excellent deformability, absorption and retention of exudates, preventing wound infections [25].

The study set out to disclose and enhance manna properties (in a drug delivery system) for pharmaceutical applications as a wound healing complex with antimicrobial and reparative activity on infected tissues. From a cosmetic point of view, considering the correlation between antioxidant skin whitening and antiaging potential, the extracts were assessed in regard to anti-tyrosinase and anti-elastase activities. The total phenolic content of each extract was also determined and a safe profile by in vitro cytotoxicity studies was verified.

## 2. Results and Discussion

**Figure 1:**
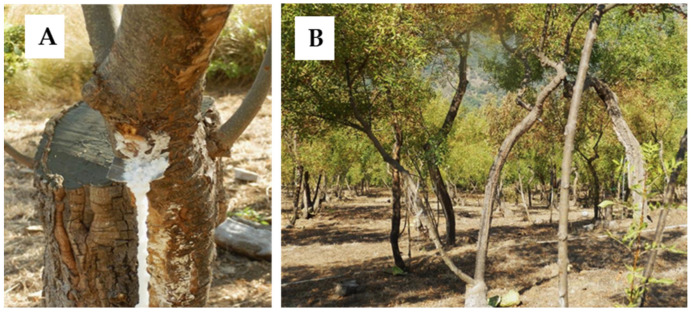
(**A**) Manna in cannoli extracted from a *Fraxinus ornus* tree plantation (**B**) located in Madonie Mountains in northern Sicily.

Figure 1 shows the collection of manna exudate taken from a *Fraxinus ornus* plantation located in the Madonie Mountains in northern Sicily. Manna exudate is obtained in specific zones where the climate in the summer is dry, hot and steadily windy. These conditions are essential for the osmotic accumulation of sugars in the plant tissues and to obtain complete drying of the manna exudate that comes out of cuts made manually on the bark.

**Figure 2:**
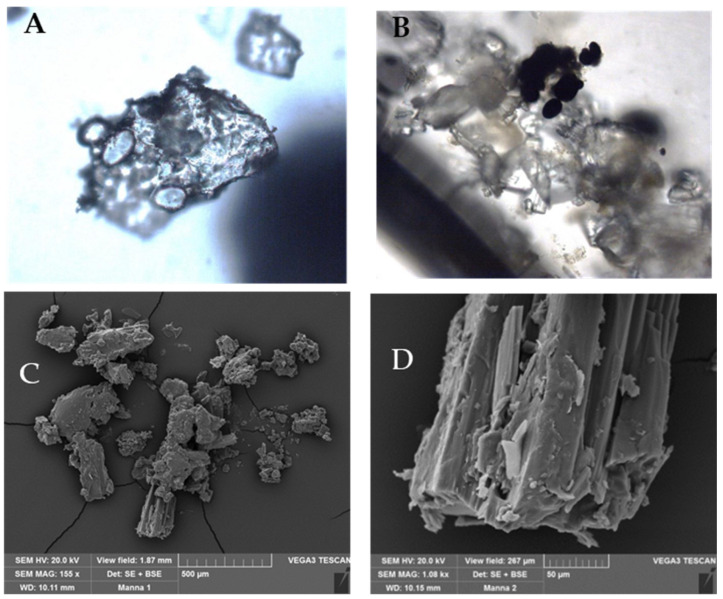
Manna samples images obtained by Light microscopy (**A**,**B**) and Scanning Electron microscopy, SEM (**C**,**D**).

Microscopic analysis was performed on the extraction of the manna exudate showing the crystal structure that is visible under both the optical and scanning electron microscopes. In Figure 2, some starch grains are visible (A); after staining with Lugol solution, starch grains become dark blue (B). In C and D, manna crystals are shown by SEM at different magnifications.

The proximate composition of manna is reported in Table 1. Data refers to the quantification of mannitol, simple sugars, and total starch content obtained by specific assays as described in Section 4.3.1.

As stated in the literature, mannitol results as the most abundant saccharide in manna extract samples, followed by smaller amounts of fructose, glucose and starch. Moreover, the total polyphenolic amount is high and comparable to the phenolic content contained in extra virgin olive oils, in agreement with the chemotaxonomic proximity between the genera *Fraxinus* and *Olea* [26].

**Figure 3:**
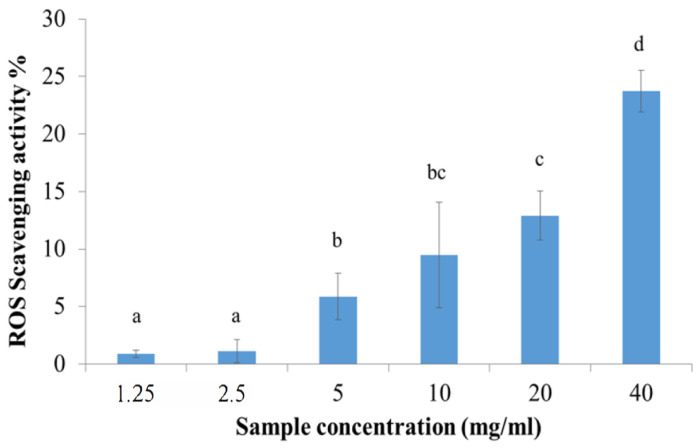
ROS-scavenging activity of manna as a function of sample concentration. Data are represented as the mean of activity percentage and related standard deviation. Different letters were chosen to indicate significant differences among groups (*p* < 0.05).

Manna extract presented a dose-dependent ROS-scavenging activity percentage (Figure 3). In particular, the best results were obtained testing manna extract at the concentration 40 mg/mL, which presented a mean antioxidant activity of 23.71 ± 1.81%.

The strong free-radical-scavenging activity and the tyrosinase-inhibiting properties seem to boost proportionally with the antioxidant amounts.

In Table 2, data related to the anti-tyrosinase activity data of manna extract are reported. Overall, manna extract exhibited low anti-tyrosinase activity. A significant effect of sample concentration (*p* < 0.05) was disclosed by the statistical analysis; in particular, samples with 20 and 40 mg/mL concentrations showed the highest inhibitory activity without any significant differences between them (*p* > 0.05).

Km and Vmax values are not influenced by the sample concentration, without any significant differences concerning negative control data.

Table 3 reports the results of anti-elastase activity at different concentrations. The data obtained reveal a good anti-elastase activity. Sample concentration significantly influenced the elastase inhibition (*p* < 0.05): from 1.25 to 10 mg/mL manna showed an activity of about 10%; considering the two highest tested concentrations, we observed a 25% inhibitory activity.

Km values of the manna sample are significantly higher with respect to negative control, while no significant differences were revealed for Vmax results. This suggests that manna extract inhibits elastase with a competitive mechanism, binding the enzyme’s active site.

**Figure 4:**
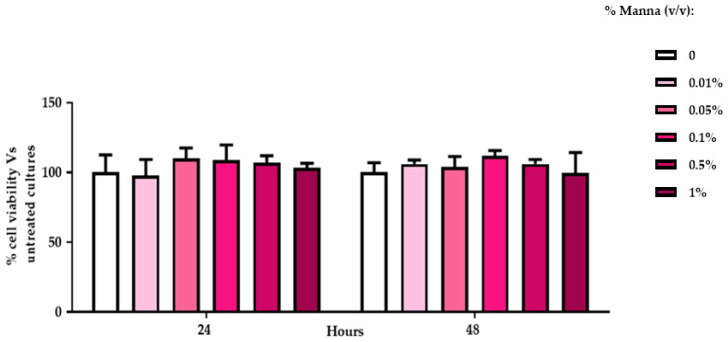
Viability index of HaCaT cells exposed to manna solution for 24 and 48 h. MTT assay was performed in HaCat exposed to different concentrations of manna solution for 24 and 48 h. MTT viability index is reported as a percentage of viability of the treated cultures vs. untreated ones. Data represent the mean ± SD of two independent experiments, performed in triplicate (ANOVA and Dunnett’s test).

Regarding cytotoxicity, all tested concentrations of manna did not evidence any toxic effects on HaCaT viability, extrapolated by MTT index (Figure 4).

The manna extract was able to increase the proliferative indices of both cell lines (HaCaT and DLD-1), especially at 48 h of treatment. After 24 h exposure to manna (0.01%, 0.05%, concentrations tested), HaCaT cells showed an inversely proportional reduction at the doses of the proliferative indices.

**Figure 5:**
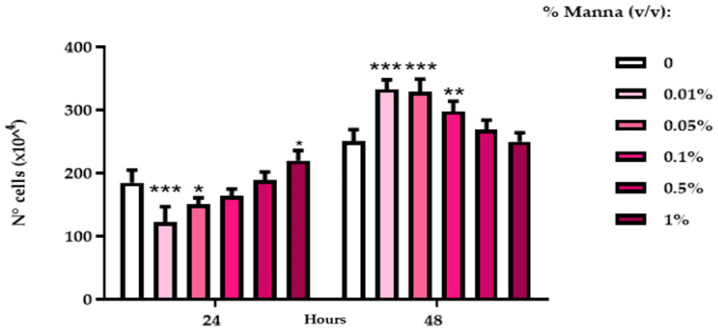
Cell proliferation indices of HaCaT cells after exposure to liquid manna formulation. The proliferation index was extrapolated from the values of the spectrofluorimetric readings of the samples submitted to the DNA Assay, referred to as a standard curve of scalar cell concentrations, performed at each test. Data represent the mean ± SD of two independent experiments, performed in triplicate.*** = *p* < 0.001, ** *p* < 0.01, * = *p* < 0.05 vs. the respective control (Two-Way ANOVA).

After 24 h exposure to manna solutions, a significant reduction of HaCaT proliferation was observed at a lower tested dose (0.01%), while at higher doses, these values increased to those of control cultures. After 48 h, manna induced a significant increase of HaCat proliferation with a dose-inverse trend (Figure 5).

**Figure 6:**
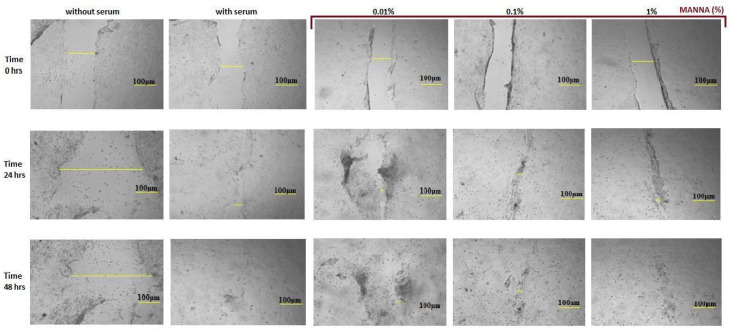
Digital images represent the incision and migration phases in untreated and manna-treated HaCaT cultures. HaCat cells were exposed to 0.01, 0.1 and 1% of manna formulation for 24 and 48 h. At each endpoint time, images of the cultures were acquired at 5× magnification. Scale bar = 100 μm.

The results obtained by scratch wound assay showed no tissue repair in negative control HaCaT cultures. The positive control cultures showed a complete recovery within 48 h in HaCaT cells exposed to manna solutions at different concentrations, progressive wound healing was observed in a dose-dependent way, and in 1% manna-exposed cultures, an almost complete injury closure was achieved (Figure 6).

**Figure 7:**
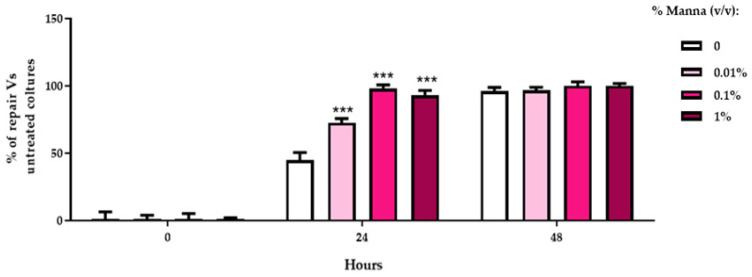
Percentage of lesion reduction in HaCaT cells exposed to the different doses of manna in liquid formulation (0.01–0.1–1%). The values, relating to the actual distances between the edges of the incision during the migration phases, were expressed in % of reduction with respect to the corresponding value measured at time 0. The images were analyzed using the ImageJ analysis software and the values represent an average of 2 determinations run in duplicate ± SD. *** = *p* < 0.001 vs. respective positive control (ANOVA and Dunnett’s test).

To quantify the data extrapolated by ImageJ 2.15.1, the percentage of lesion reduction (Figure 7) was elaborated in HaCaT cells exposed to the different concentrations of the manna solution using the formula:

100 − (mean T_48_ ∗ 100)/mean T_0._

Considering the results as a whole, it may be possible to conclude that manna solutions did not impair the mitochondrial compartment, since the MTT test did not evidence any toxic effects.

The absence of cellular suffering was also confirmed by DNA assay, and it is relevant to highlight that at the lowest tested doses (0.01 and 0.05%) of manna solutions induced an increase in HaCaT proliferative indexes. This data seems to confirm the regenerative potential of manna treatment as shown by the scratch wound assay.

Hydrogels, obtained by loading manna extract, were characterized according to pH and rheology measurements, DPPH, and Folin–Ciocalteu assays to evaluate the presence of polyphenolic compounds and the support of manna antioxidant activity when formulated in pectin hydrogels.

The pH measurements on the hydrogel samples, carried out by a pH meter, are within the normal acceptable pH range for the skin, from 5.50 to 6.80, as shown in Table 4.

The evaluation of the flow properties of a hydrogel is important for predicting an in vivo behavior, particularly regarding the spreading and coating of the formulation over the epidermis.

**Figure 8:**
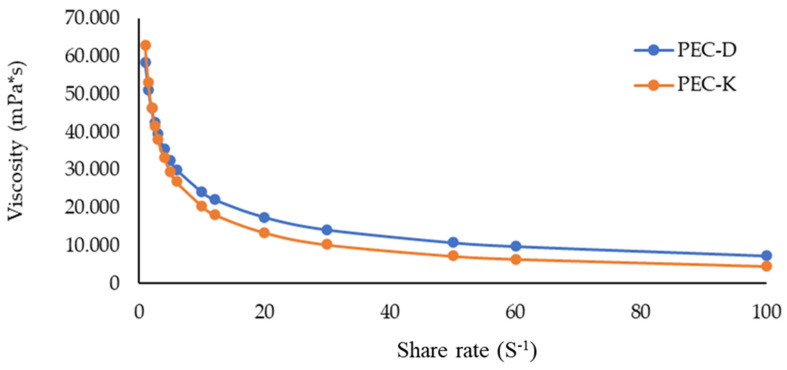
Curve of viscosity vs. shear rate of PEC-D and PEC-K.

Figure 8 depicts the viscosity curves of the two prepared gels containing manna measured at room temperature. Both formulations showed a non-Newtonian flow, and the PEC-D is more viscous than PEC-K. Anyway, for both gels, the η decreased for a small increase in γ, this trend was found up to γ approximately equal to 20. Then the viscosity did not change significantly and remained constant up to values of γ equal to 100. A behavior of this type, i.e., non-Newtonian fluids that show a decreasing viscosity as the shear rate increases for high shear rate values are defined as shear-thinning fluids having flow behavior index values *n* < 1. On the contrary, those fluids which for high values of γ present a viscosity that grow with increasing shear rate are called shear thickening dilatant fluids, having *n* > 1.

For values of γ up to 100, PEC-D and PEC-K behave as shear-thinning fluids.

The assay for the determination of the antioxidant activity (AA%) was carried out using the DPPH assay on prepared gels and the results were reported in Table 5, while the total polyphenolic (TP) content is reported in Table 6 as the result of the Folin–Ciocalteu determination.

The antioxidant activity of manna-pectin hydrogels slightly decreased when compared to manna exudate solution alone (Table 5 and Figure 3 respectively), probably due to some degradation of TP during storage at room temperature. However, the maintenance of general good antioxidant activity in the hydrogel gives us hope for possible in vivo application on the skin both as a regenerative potential of the tissues and to perform a lightening action, a prerogative sought in a cosmetic preparation.

## 3. Conclusions

The main aim of this research was to evaluate the in vitro biological activity of manna exudate to be applied as a hydrogel in the cosmetic or dermatological field. In this regard, the human keratinocyte cell line (HaCaT) was used to evaluate cytotoxicity, antiproliferative and antioxidant activities. No toxic effects were highlighted at the mitochondrial compartment level and the DNA assay confirmed the absence of a proliferative effect. The results obtained with the Scratch Assay show that already after 24 h, there is a significant lesion repair in the cell cultures exposed to different manna concentrations. From the data obtained by the radical-scavenging, anti-tyrosinase and anti-elastase tests, it seems clear that manna, when appropriately delivered in a pectin hydrogel, can prevent oxidative stress and exert an anti-inflammatory activity.

The outcomes of the study seem to indicate that manna dry exudate can be used as a natural plant bioactive ingredient in topical formulations with antioxidant properties. Pectin hydrogels can be applied as a basis for the development of new preparations used in skincare and for the treatment of skin diseases, particularly for repairing infected wounds.

## 4. Materials and Methods

### 4.1. Plant Material

Five different samples of manna in cannoli (Figure 1A) were obtained from *F. ornus* plantations located in Castelbuono (Madonie Mountains, Palermo, Italy) (Figure 1B). The samples were supplied by different producers in August 2019. Voucher specimens were deposited at the Department of Drug Science of the University of Pavia (Italy). Manna samples (50 g) were then completely dissolved in 50 mL distilled water and kept at room temperature in the dark for 1 h. The extracts were obtained by Büchner funnel filtration through a 0.45 μm filter (Millipore, Billerica, MA, USA), subsequent lyophilization, and stored at −80 °C.

### 4.2. Microscopic Characterization

Small manna fragments in cannoli were dissolved with a few drops of 70% ethanol and then observed by transmission-light microscope (Leica DM 2000—Leica Microsystems, Wetzlar, Germany), equipped with a Leica DFC 320 camera A Lugol’s iodine solution (iodine and potassium iodide water solution, 1:2) was used as the reactive to assess the presence of starch in the samples. For Scanning Electron Microscope (SEM) analyses, manna fragments dissolved in 70% ethanol were placed on aluminum stubs using carbon adhesive tabs, coated with 10 nm gold, and observed by a scanning electron microscope at an accelerating voltage of 20 kV (Vega3 Tescan LMU SEM) (Tescan USA Inc., Cranberry Twp, PA, USA).

### 4.3. Phytochemical Screening

#### 4.3.1. Quantification of Mannitol, Sugars and Total Starch Content

Mannitol, gucose, fructose, and total starch content were quantified by spectrophotometric analysis (Jasco V550 spectrophotometer, Jasco, MD, USA) at 340 nm, using specific Megazyme^®^ assay kits that allow the analyte quantification, exploiting reduction reactions using the mean of specific enzymes. Spectral data collected were then reported on a Megazyme^®^ Mega-Calc™, a datasheet developed to assist in calculating the concentration of analyte from raw absorbance data.

Mannitol quantification was assessed by K-MANOL™ kit on aqueous solutions of the sample (1 mg/mL), evaluating NAD+ reduction into NADH. Cuvettes were prepared as follows: 1 mL of MilliQ water, 0.05 mL of sample solution, 0.05 mL of phosphate buffer and 0.05 mL of NAD^+^. After 2 min incubation, the mixture was analyzed, and absorbance was recorded. Then, 0.01 mL mannitol dehydrogenase (ManDH) was added; absorbance was monitored until the end of the reduction reaction, every two minutes, and raw spectral data was processed as described above. Mannitol was expressed as a percentage amount (*w*/*w*) of the dry powder.

Glucose and Fructose were quantified by K-FRUGL™ kit. This kit allows for simultaneous quantification, exploiting the reaction of hexokinase (HK), glucose-6-phosphate dehydrogenase (G6P-DH) and phosphoglucose isomerase (PGI) by measuring NADPH amounts in samples after an enzymatic treatment. Cuvettes were set as follows: 1 mL of MilliQ water, 0.05 mL of sample aqueous solution (1 mg/mL), 0.05 mL of phosphate buffer and 0.05 mL of NADP^+^/ATP. Absorbance was read after about 3 min of incubation against a blank without the sample. Then, 0.01 mL of HK/G6P-DH was added to start the reaction to evaluate glucose. After 5 min, absorbance was read. The last step was the addition of 0.01 mL of PGI to quantify fructose; after 10 min, absorbance is read. Glucose and fructose are expressed as a percentage amount (*w*/*w*) of the dry powder.

Total starch content (TSC) was evaluated by a K-TSHK™ assay Kit. The TSC content was quantified by measuring NADPH amounts in samples after the opportune enzymatic treatment. An amount of 50 mg of dry powder was added to 0.2 mL ethanol 80% *v*/*v* and 1 mL of a 2M KOH solution, stirring the solution with a magnetic stirrer for 20 min at 4 °C. Subsequently, 4 mL of a sodium acetate buffer solution (1.2 M, pH 3.8) was added, followed by 50 μL of α-amylase (8300 U/mL) and 50 μL of amyloglucosidase (AMG, 3300 U/mL). Samples were kept at 50 °C for 30 min, vortexing every 5 min with subsequent centrifugation at 3000 rpm for 10 min, to recover the supernatant. Reagents were mixed in a quartz cell at the following amounts: 1 mL H_2_O, 25 μL supernatant, 50 μL phosphate buffer solution pH 7.6, 50 μL NADP+/ATP. The solution was kept at room temperature for 3 min; then, the absorbance was recorded at 340 nm against the blank containing water instead of the sample. Then, 10 μL of a solution containing hexokinase (HK) and glucose-6-phosphate-dehydrogenase (G6P-DH) was added. After 5 min at room temperature, absorbance was recorded again against the blank. Absorbance values were reported on the Mega-Calc sheet for quantification. TSC is expressed as a percentage amount (*w*/*w*) of the dry powder.

#### 4.3.2. Quantification of Manna Phenolic Compounds

Total polyphenols (TPs) in manna exudate were quantified by the Folin–Ciocalteu assay, a spectrophotometric test exploiting the reaction of the sample with phosphomolybdate and phosphotungstate salts. A calibration curve was made with gallic acid as the reference standard, ranging from 10 mg/L to 100 mg/L. Cuvettes were set as follows: 400 µL MilliQ water, 80 μL standard of samples, 40 µL of Folin–Ciocalteu reagent and 480 µL of 10.75% Na_2_CO_3_ solution. After 30 min of incubation, absorbances were acquired at 760 nm using a Jasco V550 spectrophotometer [27,28].

Tannin content was evaluated with a spectrophotometric assay suggested by Porter et al. (1985) [29] which consists of a sample treatment with n-Butanol:12N HCl:FeCl_3_ (10:10:3 *v*/*v*/*w*) as the reagent. The sample was diluted in MilliQ water at a 2 mg/mL concentration. For each sample, two tubes set with 300 µL of sample + 900 µL of reagent were used. One tube was incubated at 100 °C for 30 min, the other one at room temperature for the same amount of time. Then, 1 mL of each prepared solution was put into the cuvette, and absorbances were recorded at 550 nm by a Jasco V550 spectrophotometer. Tannin content was calculated as the difference between the absorbances (A sample at 100°—A sample at RT), multiplied by a constant [29].

#### 4.3.3. Manna Radical Scavenging Activity

The antioxidant activity of manna exudate [30] was assessed by the 2,2-diphenyl-1-picrylhydrazyl (DPPH) colorimetric test. DPPH was solubilized in methanol (Sigma Aldrich (Merck Spa), Milan, Italy, 2.8% *w*/*v*) and added to aqueous manna solutions at different concentrations (1.25, 2.5, 5, 10, 20 and 40 mg/mL), maintaining a 1:9 (DDPH:manna) volume ratio. The reaction mixture was kept in the dark at room temperature and centrifuged at 3000 r/min for 5 min. Supernatants were analyzed at 515 nm using a Synergy, BioTek spectrophotometer. The sample absorbance values were subtracted from the blank ones (reaction mixture without DPPH). As the control, the complete reaction mixture without manna was considered. Each experiment was performed in triplicate and results were processed by the ANOVA model, considering the sample concentration as a fixed factor. The radical-scavenging activity was calculated using the following formula:

% activity = [(Actr − Asamp)/Actr] ∗ 100

where Actr = control absorbance and Asamp = sample absorbance.

#### 4.3.4. Anti-Tyrosinase Activity

The anti-tyrosinase activity [30] of the manna sample was evaluated by analyzing the reaction kinetic between the enzyme tyrosinase and its L-tyrosine substrate, according to the Michaelis–Menten model. Tyrosinase (Sigma Aldrich 500 IU/mL) solubilized in phosphate buffer (pH 6.8) was incubated with manna samples (1.25, 2.5, 5, 10, 20 and 40 mg/mL) for 10 min. Then, a L-tyrosine solution (Sigma Aldrich) was added to the reaction mixture. The reaction kinetics were analyzed at the wavelength of 480 nm for 30 min (Synergy, BioTek). As the negative control, the reaction mixture without a sample was used, while an arbutin solution (Sigma Aldrich) was used as a positive control. All sample absorbance data were subtracted from the absorbance value of the reaction mixture containing manna (without any enzyme and substrate). Each analysis was conducted in triplicate. The anti-tyrosinase activity percentage was calculated using the formula:

% activity = [(Actr − Asamp)/Actr] ∗ 100

where Actr = control absorbance; Asamp = sample absorbance

#### 4.3.5. Anti-Elastase Activity

A spectrophotometric analysis of the kinetic reaction between elastase and N-Succinyl-Ala-Ala-Ala-p-Nitroanilide substrate [30] was used to evaluate the ability of manna samples to inhibit the elastase enzyme activity. Briefly, a pancreatic porcine elastase solution (Sigma Aldrich, 0.5 IU/mL in phosphate buffer pH 6.8) was co-incubated with manna samples (1.25, 2.5, 5, 10, 20 and 40 mg/mL) for 20 min. Then, N-Succinyl-Ala-Ala-Ala-p-Nitroanilide substrate (Sigma Aldrich, 0.41 mM in TRIS buffer) was added to the reaction mixture, monitoring the kinetic reaction at 410 nm for 35 min by spectrophotometric analysis (Synergy, BioTek, Agilent Technologies Italia Spa, Milan, Italy), according to Michaelis–Menten kinetics. As a negative control, the reaction mixture without a sample was used, while the epigallocatechin gallate (Sigma Aldrich) was selected as a positive control. All the absorbance values related to the sample were subtracted from the absorbance of the blank mixture, composed only of the sericin sample. Each analysis was performed in triplicate and the results were reported as an anti-elastase activity percentage using the formula:

% activity = [(Actr − Asamp)/Actr] ∗ 100

where Actr = control absorbance; Asamp = sample absorbance the Michaelis–Menten model was applied to process the enzymatic kinetic parameters of anti-tyrosinase and anti-elastase activities

y = (Vmax ∗ x)/(Km + x)

where y represents the absorbance at time x, Km (μM) corresponds to the time at which the activity is half of the maximum, while Vmax (μmol/min) is the maximum extrapolated rate. Kinetic data were elaborated by Graph-Pad Prism 8 software 10.2 for the Windows platform. For each curve, Vmax and Km were analyzed by an ANOVA model, considering sample concentration as a fixed factor. Differences among groups were analyzed by posthoc LSD’s test for multiple comparisons, fixing the statistical significance at *p* < 0.05.

### 4.4. In vitro Cytotoxicity Analysis

#### 4.4.1. Maintenance of Cell Culture

HaCaT human cell lines, derived from a spontaneously immortalized keratinocyte, were certified by STR DNA profile analysis by Biological Bank, a Core Facility of the IRCCS San Martino University Hospital—IST National Institute for Cancer Research (Genoa, Italy). HaCaT cells were cultured at 37 °C under 5% CO_2_ in DMEM medium added by 10% heat-inactivated FBS serum. To avoid any potential interference with the experimental conditions, neither antibiotic nor anti-fungine solutions were added to the standard or experimental medium. Medium was replaced every 2/3 days and cells were sub-cultured by TripLE™ Express (Invitrogen Life Technologies, Van Allen Way Carlsbad, CA, USA) treatment when the original flask was about 75% confluent. Each cell culture was assessed as mycoplasma-free during each regular control with the Reagent Set Mycoplasma Euroclone (Euroclone, Milan, Italy). Cells were seeded in 96, 12, and 48 well plates at 20, 100, 40 × 10^3^ cells/well, respectively, 24 h before MTT, DNA and Scratch assay, respectively. To carry out the analysis, 24 h after seeding, the HaCaT cells were exposed to different concentrations of manna master stock solution (0.002 mg/mL distilled sterile water) for 24 and 48 h. At each checkpoint time, cell cultures were submitted to the selected analysis. This analysis included three independent experiments, with each sample being tested in triplicate.

#### 4.4.2. MTT Assay

Cell viability was assessed at the end of each experimental treatment by thiazolyl blue tetrazolium dye reduction assay (MTT) [31]. The optical densities (OD) of the dissolved formazan crystals (for MTT test) were evaluated at 570 nm by spectrophotometric analysis. Compounds were considered toxic when cell viability was reduced by 15%, compared to untreated cultures, according to the ECVAM’s guidelines testing any cytotoxic effects of the compounds and in parallel for measuring cell proliferation, according to the manufacturer’s instructions.

#### 4.4.3. DNA Assay

Cell proliferation was analyzed regarding DNA content using a Hoechst 33,258 solution [32,33]. At a fixed time point, the experimental medium was removed and then cells were exposed to a lysis solution (urea 10 M, 0.01% SDS in saline sodium citrate buffer, SSC), after washing with warm PBS. The dissolved cell suspensions were kept for 2 h at 37 °C in a shaking bath. Subsequently, 1 mL of Hoechst 33,258 dye solution (1 mg/mL in SSC buffer) was added in the dark. Dye absorbance was spectrofluorometrically measured at excitation and emission wavelengths of 355 nm and 460 nm, respectively. Cell proliferation was calculated by referring fluorescence units to a linear standard curve for DNA fluorescence versus cell number, by using a range of split cells from 60 to 100 × 10^3^ cells in duplicate and then stored at −20 °C until the execution of the assay on the samples of the treated cells.

#### 4.4.4. Scratch Wound Assay

After reaching a semi-confluence state (48 h from seeding), HaCaT were subjected to serum starvation and left overnight in a humidity-controlled incubator at 37 °C, 5% CO_2_. Thereafter, the medium was removed, and monolayers were washed with PBS. Then, a scratch wound was simulated in the middle of the monolayer, by creating a gap using a sterile 10 µL pipette tip. Cell debris was washed away using PBS and subsequently, cells were exposed to manna solutions (0.01, 0.1, and 1%, *v/v* in complete media plus FBS). In parallel, negative and positive controls were performed by growing cells in medium, *w*/*o* and 10% FBS, respectively. All treatments were performed in 3 experiments run sixfold. Wound closure was monitored at 0, 24, 48 h with inverted phase contrast microscopy, and images (5× magnification) were captured to be submitted at the following quantification of the wound healing. The images were elaborated by an open-source platform for biological image analysis (Fiji-ImageJ software). The ratio of open areas at 24 h to open areas at 0 h was determined and replicates were averaged. On day 0 (0 h), scratches were designed as 100% open and used as the reference to calculate the % closure for all scratches, in terms of left distance over time of cell migration into the scratch wound area. For Scratch tests: significance was assessed by a two-way analysis of ANOVA variance followed by Dunnett’ test, as described in figures, using GraphPad Prism 5.03 for Windows and GraphPad Software, (GraphPad Inc., La Jolla, CA, USA). Statistically significant differences were fixed at *p* < 0.01.

### 4.5. Hydrogel Preparation

Hydrogels of citrus pectin (2.5% *w*/*w*) loaded with manna (1% *w*/*w*) and containing a preservative system to prevent bacterial growth were prepared by dispersing the required amounts of pectin (PEC) and manna in 10 mL of deionized water, under constant stirring and adding two different types of preservative: 2% *w*/*w* Dermosoft 1388^®^ (D) (Evonik Dr. Straetmans GmbH, Hamburg, Germany) and 0.5% *w*/*w* potassium sorbate (K), respectively. Dermosoft 1388^®^ is a convenient blend of glycerin, water, sodium levulinate and sodium anisate that offers an effective and synergic antimicrobial activity. So that gelation occurs, 1 mL calcium chloride (CaCl_2_), 1% solution, is added dropwise. The samples obtained were marked with the acronyms PEC-D and PEC-K, according to the added preservative. The respective gels without manna were also prepared (BN PEC-D and BN PEC-K). All gel samples were stored at 20 °C for 24 h before use. The composition of hydrogel formulations is disclosed in Table 7.

### 4.6. Hydrogel Characterization

#### 4.6.1. pH Measurement

pH values of studied hydrogels were determined by a digital pH meter (calibrated before use with pH 4.0 and 7.0 standard buffers) dissolving 1 g gel in 100 mL of distilled water and stored for 2 h [34] at room temperature.

#### 4.6.2. Rheological Study

The rheological behavior of manna formulations was studied by a continuous shear method using a Brokfield viscometer, Viscostar-R (FUNGILAB S.A.), Turin, Italy. An amount of 50 mL of each sample was subjected to shear rates in a range from 1 to 100 s^−1^. All measurements were carried out at room temperature.

#### 4.6.3. Gel Antioxidant Activity

Hydrogel antioxidant activity was evaluated by the DPPH assay [35], based on the discoloration rate of the stable free radical 2,2-Diphenyl-1-picrylhydrazyl (DPPH) after reaction with the antioxidant agent. An opportune gel amount (0.5 g) was dissolved in 5 mL water and then a 0.1 mL aliquot of the obtained solution was mixed with 3.9 mL of DPPH methanolic solution (65 µM). After storage for 30 min in the dark, absorbance was detected at 516 nm. A linear calibration curve was obtained using Trolox as the standard (concentrations ranging between 20 to 200 mg/L, R^2^ = 0.9955). Results were calculated as Trolox equivalents in mg/L, and the percentage of antioxidant activity (AA%) was calculated by the equation:

AA% = [(A0 − As)/A0] × 100

Corresponding to the ratio of decreasing absorbance of sample solution (A0 − As) to DPPH solution absorbance of blank (A0).

#### 4.6.4. Folin–Ciocalteu Spectrophotometric Determination in Gels

The total polyphenols (TPs) content in manna hydrogels was evaluated by the Folin–Ciocalteu assay (FC) [36]. The Folin–Ciocalteu reagent consists of a yellow mixture of sodium phosphotungstate and phosphomolybdic acid capable of oxidizing polyphenolic substrates and other antioxidant molecules, giving the solution an intense blue color measured spectrophotometrically at 760 nm. The intensity increases linearly with the concentration of polyphenolics in the reaction medium TPs amount, and was extrapolated from a calibration curve prepared with gallic acid standard solutions ranging from 20 to 80 mg/L (R^2^ = 0.9988); values were expressed as mg of gallic acid equivalents per g (GAE/g) of gel.

### 4.7. Statistical Analysis

Radical-scavenging, anti-tyrosinase, anti-elastase activity and scratch test results were processed by an ANOVA variance analysis, considering sample concentration and time (for enzymatic inhibitory activities) as fixed factors. The differences between groups were analyzed with the posthoc LSD’s test for multiple comparisons. Data are expressed as means of triplicate data ± standard deviation. The statistical significance was fixed at *p* < 0.05.

## Figures and Tables

**Table 1:** Summary of phytochemical screening expressed in g/100 g of lyophilized manna extract. (Data represent the mean values of triplicate determinations ± SD).

Compound	Quantity (g/100 g)
Glucose	2.43 ± 0.04
Fructose	5.84 ± 0.27
Resistant Starch	1.93 ± 0.08
Starch	1.34 ± 0.08
Mannitol	75.78 ± 0.68
Total polyphenols	0.21 ± 0.03
Hydroxycinnamic Acids	0.04 ± 0.01
Flavonoids	0.02 ± 0.01
Tannins	0.06 ± 0.03

**Table 2:** Anti-tyrosinase activity of manna extracts as a function of concentration. Data represents the overall mean and standard error (SE) of activity percentage, Km and Vmax. CTR: negative control. Different letters superscripts refer to significant differences within each considered parameter (*p* < 0.05).

Sample Concentration (mg/mL)	Anti-Tyrosinase Activity % (Mean ± SE)	Km (μM) (Mean ± SE)	Vmax (μmol/min) (Mean ± SE)
CTR	-	19.75 ± 0.645 ^a^	1.43 ± 0.022 ^a^
1.25	0.46 ± 0.069 ^a^	25.47 ± 1.041 ^a^	1.58 ± 0.034 ^a^
2.5	2.33 ± 1.122 ^a^	19.92 ± 0.843 ^a^	1.43 ± 0.029 ^a^
5	3.63 ± 1.401 ^a^	20.29 ± 0.634 ^a^	1.45 ± 0.022 ^a^
10	5.47 ± 1.152 ^a^	19.91 ± 0.632 ^a^	1.45 ± 0.022 ^a^
20	10.08 ± 3.584 ^b^	19.54 ± 0.949 ^a^	1.42 ± 0.033 ^a^
40	10.96 ± 4.051 ^b^	18.78 ± 0.884 ^a^	1.31 ± 0.029 ^a^

**Table 3:** Anti-elastase activity of manna extract as a function of concentration. Data are represented as the overall mean and standard error (SE) of activity percentage, Km and Vmax. CTR: negative control. Different letters superscripts refer to significant differences within each considered parameter (*p* < 0.05).

Sample Concentration (mg/mL)	Anti-Elastase Activity % (Mean ± SE)	Km (μM) (Mean ± SE)	Vmax (μmol/min) (Mean ± SE)
CTR	-	6.63 ± 0.387 ^a^	0.98 ± 0.016 ^a^
1.25	10.69 ± 4.086 ^a^	12.57 ± 4.166 ^b^	0.87 ± 0.112 ^a^
2.5	10.16 ± 3.140 ^a^	8.45 ± 0.389 ^b^	1.06 ± 0.016 ^a^
5	11.57 ± 2.772 ^a^	8.48 ± 0.287 ^b^	1.03 ± 0.011 ^a^
10	16.13 ± 3.003 ^a^	8.18 ± 0.525 ^b^	1.00 ± 0.020 ^a^
20	26.00 ± 3.452 ^b^	11.63 ± 0.456 ^b^	1.08 ± 0.016 ^a^
40	25.05 ± 5.110 ^b^	12.16 ± 0.794 ^b^	1.11 ± 0.028 ^a^

**Table 4:** pH values for the prepared hydrogels, mean value ± standard deviation (SD) of three independent experiments (*n* = 3).

Hydrogel Sample	pH
BN PEC-D	5.04 ± 0.02
BN PEC-K	6.10 ± 0.01
PEC-D	5.50 ± 0.01
PEC-K	6.80 ± 0.02

**Table 5:** Antioxidant activity percent (AA%) obtained by DPPH assay and expressed as mean values ± SD of three independent experiments (*n* = 3).

	Absorbance λ = 515 nm	% DPPH	AA%
**DPPH**	0.443	100	-
**PEC-K**	0.340	76.75 ± 0.3	11.71 ± 0.3
**PEC-D**	0.351	79.23 ± 0.2	10.33 ± 0.2

**Table 6:** Total phenolic content obtained by Folin–Ciocalteu assay and expressed as mean values ± SD of three independent experiments (*n* = 3).

	Absorbance λ = 750 nm	mg GAE/g gel
**PEC-K**	0.436	35.66 ± 0.15
**PEC-D**	0.361	29.51 ± 0.22

**Table 7:** Composition of different prepared hydrogel formulations.

Formulation	Pectin Citrus (% *w*/*w*)	Manna (% *w*/*w*)	CaCl_2_ (mL)	Dermosoft (%)	Potassium Sorbate (%)
BN PEC-D	2.5	-	1	2	-
BN PEC-K	2.5	-	1	-	0.5
PEC-D	2.5	1	1	2	-
PEC-K	2.5	1	1	-	0.5

## Data Availability

The raw data supporting the conclusions of this article will be made available by the authors on request.
